# Alpine steppe vegetation communities are more sensitive to plateau pika disturbance than alpine meadows

**DOI:** 10.3389/fpls.2025.1546828

**Published:** 2025-05-29

**Authors:** Rui Hua, Peng Zhang, Liqing Wang, Miaomiao Huang, Limin Hua, Jianwei Zhou

**Affiliations:** ^1^ Institute of Grassland Research, Chinese Academy of Agricultural Sciences, Inner Mongolia Key Laboratory of Grassland Conservation Ecology, Hohhot, China; ^2^ Monitoring and Planning Institute of Inner Mongolia Forestry and Grassland Administration, Ecological Restoration Section, Hohhot, China; ^3^ College of Grassland Science, Gansu Agricultural University, Engineering and Technology Research Center for Alpine Rodent Pest Control of National Forestry and Grassland Administration, Lanzhou, China

**Keywords:** alpine steppe, alpine meadow, plateau pika, vegetation community response, interference threshold

## Abstract

**Introduction:**

Small herbivores are important biological factors affecting plant productivity and species richness in the grassland ecosystem of the Tibetan Plateau. However, the response of different grassland types to the disturbance of the “endemic species” plateau pika (*Ochotona curzoniae*) remains unclear.

**Methods:**

In this study, two representative grassland types, alpine meadow and alpine steppe, were taken as research objects to compare the effects of pika disturbance on vegetation structure and function indicators, and to explore the disturbance response threshold of these ecosystems.

**Results:**

The results showed that the alpine steppe was more sensitive to pika disturbance, with significant decreases in biomass, vegetation height, and coverage even at low disturbance levels. In contrast, alpine meadows exhibited greater resilience, maintaining higher productivity and species diversity under moderate disturbance conditions. Notably, the functional index of alpine meadow peaked under moderate disturbance, while alpine steppe rapidly transitioned to a degraded state. These differences highlight the varying disturbance thresholds between the two grassland types.

**Discussion:**

This study highlights the vulnerability of alpine grassland ecosystems to pika disturbance and provides a strong scientific basis for designing effective grassland management and ecological restoration strategies.

## 1 Introduction

With the intensification of global climate change, the stability and functioning of ecosystems are facing unprecedented challenges (Lennox and Gowdy, 2014). This is particularly true in alpine regions, where unique climatic, edaphic, and biological conditions create highly fragile ecological structures. As a critical component of global ecological balance, alpine grassland ecosystems serve as sensitive indicators of climate change and support rich biodiversity, making them of significant ecological conservation value (Nikolaishvili, 2015). However, these ecosystems are increasingly subjected to multiple stressors, including climate change, land-use modifications, and biological invasions, which collectively threaten their ecological integrity (Wei et al., 2020). Such cumulative pressures are profoundly altering the ecological functions of alpine grasslands and exacerbating their inherent vulnerability, highlighting the importance of the “United Nations Decade on Ecosystem Restoration” (2021–2030) initiative, which aims to improve the stability and resilience of global ecosystems by restoring damaged and degraded ecosystems (United Nations Environment Programme (UNEP) & Food and Agriculture Organization of the United Nations (FAO), 2021).

Alpine meadow and steppe are the two primary types of alpine grasslands, and they exhibit distinct differences in vegetation characteristics, ecological environments, and biodiversity (Sun et al., 2023). And the growing season length of alpine steppe is shorter than that of alpine meadow (Wang et al., 2006). Alpine steppe is characterized by sparse perennial herbs, nutrient-poor soils, and relatively low biodiversity (Dong et al., 2020). In contrast, alpine meadow supports greater plant higher moisture levels and biodiversity (Zhang et al., 2016). Biodiversity has been widely recognized as a core indicator of ecosystem health (Vira and Adams, 2009), with higher biodiversity conferring greater resilience to disturbances, such as herbivory, by sustaining a broader range of ecological functions. Conversely, ecosystems with low biodiversity are often more vulnerable to species loss and functional degradation (Tilman et al., 1997). Thus, the recovery potential of alpine steppe and alpine meadow is closely tied to their respective biodiversity levels (Holling, 1973).

The plateau pika, a small herbivorous mammal endemic to the Qinghai-Tibetan Plateau, significantly influences vegetation communities through its burrowing and grazing behaviors (Smith et al., 2019; Zhang et al., 2016). These activities not only alter plant species composition but also impact community structure and overall ecosystem functions (Liu et al., 2013). According to the Disturbance Hypothesis, the long-term stability of ecosystems is shaped by external disturbances, with excessive disturbances potentially resulting in ecosystem degradation (Connell, 1978). When plateau pika populations reach high densities, their activities can lead to a reduction in plant diversity, accelerate grassland degradation, and compromise vital ecosystem services (Xu et al., 2024; Li et al., 2021). However, it is essential to recognize that pikas also fulfill a vital ecological role; they act as a keystone species within alpine grassland food webs and serve as “ecosystem engineers” that influence both vegetation and soil structures (Smith et al., 2019). The extent of ecosystem changes driven by pika activities largely depends on the intensity of their disturbances. Notably, despite the widespread distribution of plateau pikas in alpine meadows and alpine steppes, current plateau pikas population control and culling practices in the Tibetan Plateau region do not distinguish between these two grassland types. Instead, a uniform management standard is applied to regulate plateau pika populations across both ecosystems, which may overlook the specific ecological dynamics present in each grassland type.

Building on these premises, we hypothesize that the sensitivity of alpine steppe and alpine meadow vegetation communities to pika disturbances differs. This study aims to investigate the differential responses of alpine steppe and alpine meadow ecosystems to varying intensities of pika disturbance, thereby uncovering the potential risks that biological disturbances pose to alpine ecosystems. To achieve this goal, we will address the following key questions:

1. How do alpine steppe and alpine meadow differ in their ecological responses to various levels of plateau pika disturbance?
2. What impacts do pika disturbances have on vegetation diversity in alpine steppe and alpine meadow?
3. What is the difference between vegetation diversity and aboveground biomass in the two types of grassland?

The findings of this study will enhance our understanding of the vulnerability and recovery potential of alpine steppe and alpine meadow, providing a theoretical foundation for developing effective ecological conservation and restoration strategies in alpine regions.

## 2 Materials and methods

### 2.1 Study area

**Figure 1:**
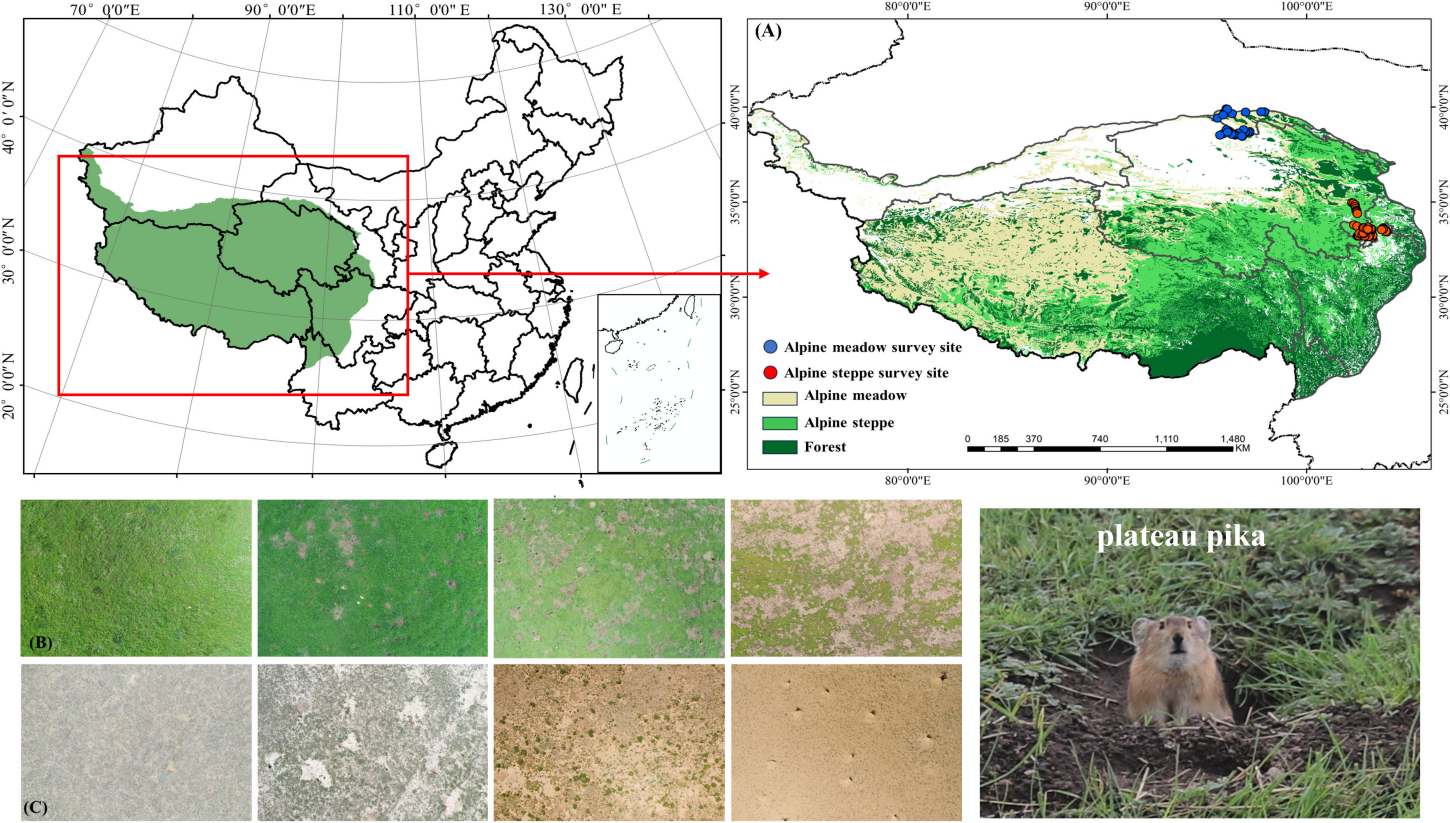
The location of the research site **(A)** and the images of alpine meadow **(B)** and alpine steppe **(C)** taken by UAV under different disturbance degree of Plateau pika.

The alpine meadow survey areas are located in the northern region of Sichuan Province on the Qinghai-Tibet Plateau (102°33′–102°59′ E, 33°48′–34°10′ N), the western region of Qinghai Province (101°7′–101°46′ E, 34°37′–35°27′ N), and the southern region of Gansu Province (101°12′–102°24′ E, 33°25′–34°28′ N), China (**Figure 1**). These areas have an average elevation of 3,000–3,600 m, an annual mean temperature ranging from -3 to 2°C, and annual precipitation of 599.5–648.5 mm. There is no absolute frost-free period, and the plant communities are dominated by *Kobresia pygmaea, Elymus nutans*, and *Anemone trullifolia.*

The alpine steppe survey area is situated in the western Qilian Mountains of Gansu Province (94°59′–97°33′ E, 38°2′–39°49′ N), with an average elevation exceeding 4,000 m. The region has an annual mean temperature of approximately 1.8°C and annual precipitation of 450–780 mm. The plant communities are primarily dominated by *Stipa purpurea* and *Poa* sp*hondylodes*.

### 2.2 Field experiment design

The study was conducted across the Qinghai-Tibet Plateau, where a total of 56 plots were established using a stratified random sampling approach: 30 plots in alpine meadow (AM) and 26 plots in alpine steppe (AS). Each plot measured 1 hectare (100 m × 100 m), with a minimum distance of 1 km between plots of the same grassland type to ensure spatial independence. To minimize confounding effects from grazing, all plots were located in flat, homogeneous winter grazing areas with consistent historical land-use patterns.

Within each plot, three quadrats were randomly placed. Quadrat sizes were adjusted to account for differences in vegetation structure: 1 m × 1 m quadrats were used in the denser alpine meadows, while 0.5 m × 0.5 m quadrats were employed in the sparser alpine steppes. For each quadrat, seven disturbance-related metrics were recorded: effective pika hole density, vegetation cover aboveground biomass, mean community height, species richness, Shannon-Wiener diversity index, and the proportion of edible grass species (identified through local pastoral knowledge and regional flora guides).

From 2017 to 2019, during the peak growing season (July–August), high-resolution aerial imagery was acquired under clear, windless conditions using a DJI Mavic 2 Pro drone equipped with a Hasselblad L1D-20c camera (20 MP sensor). The drone was flown at a consistent altitude of 20 m, achieving a ground resolution of approximately 1 cm/pixel, and followed pre-programmed routes to ensure complete coverage of each 100 m × 100 m plot with 80% image overlap. Synchronous ground surveys were conducted to validate aerial data, including precise GPS coordinates, elevation measurements, and manual vegetation cover assessments within quadrats. Additionally, potential errors in drone imagery (such as shadow effects) were minimized by conducting surveys under uniform solar conditions, with the root-mean-square error (RMSE) for key variables remaining below 8%.

### 2.3 Field survey and sampling

The vegetation cover at each plot was assessed through the interpretation of orthophotos using supervised classification in ENVI 5.1 software. To ensure the reliability of the methodology, vegetation cover derived from drone image interpretation were cross-validated against ground-based measurements, demonstrating strong correlations (**Supplementary Figure S1**). Aboveground biomass within each quadrat was measured by harvesting all vegetation, followed by drying the samples in an electric thermostatic oven at 65°C for 6 hours and weighing the dry biomass. “Plugging tunnels method” was adopted to determine the number of active burrow entrances to replace the relative population density of plateau pika. In this method, the burrow entrances were plugged with dry hay for one day and one night, and the number of plugs that were cleared by the plateau pikas to gain access to the grassland surface was recorded (Zhang et al., 2020). To assess community height and species richness, plant height was measured within quadrats, species richness was determined by randomly throwing sampling rings, edible grasses were identified, and their proportion was calculated. Based on the survey results, species richness is replaced by the total number of species in the vegetation community, the Shannon-Wiener diversity index for each plot was calculated as following:


$$\text{H}=-\sum p_{i}\times ln_{p_{i}}$$

H is the Shannon-Wiener diversity index, and Pi is the proportion of species i in the quadrat.

### 2.4 Statistical analysis

After the indicators were screened, this study adopted Principal Components Analysis (PCA) to determine the weight of each indicator in the degree of rodent disturbance (Joshi et al., 2006). grassland health index (GHI) was established to comprehensively evaluate the disturbance degree of plateau pika.


$$\text{GHI}=\sum_{i=1}^{n}v_{i}w_{i}$$

The Min-Max standardization method is used to standardize the measured values of each index. $w_{i}\;$ is the weight of each index, and principal components analysis is used to determine the weight of each index. m is the number of indicators. The differences among environmental parameters, productivity and species diversity indexes were examined by non-parametric Wilcoxon rank-sum test. In order to determine the key factors influencing species diversity in different grassland types, the richness and Shannon-Weiner diversity index were the response variables and spatial, climate, rodent disturbance and soil factors were the predictors. mantel test was conducted to determine the factors related to species diversity. All statistical analysis and plotting were done in R 4.2.3.

The relative change rates of different indexes of alpine meadow and alpine steppe were calculated, that is, the percentage change of each index relative to the lowest disturbance state under the condition of the highest pika density. The loess regression method is used to capture the local trend of vegetation change in alpine meadow and alpine steppe under different rat density. Geographic feature vectors and soil data: In order to fully characterize the geographic structure of sampling sites and consider spatial autocorrelation, this study converted the latitude and longitude information into geographic feature vectors (Borcard and Legendre, 2002). The computation of space vectors (i.e. Moran’s Eigenvector Maps, abbreviated MEM) is done by using the dbmem function in the R software package adspatial (Dray et al., 2017). MEM is an orthogonal spatial variable generation method based on eigen decomposition of spatial adjacency matrices, which quantifies and analyzes spatial autocorrelation structures by extracting multi-scale spatial eigenvectors. Soil organic matter and pH were obtained from Harmonized World Soil Database version 1.2 (HWSD V1.2) for the respective grassland types. Climate data is obtained from the China Meteorological Data Network (Data.cma.cn), and the average annual temperature and precipitation information are obtained through statistics.

## 3 Results

### 3.1 Relationship between disturbance of plateau pika and environmental factors

The comparative analysis of various indicators in the alpine steppe and alpine meadow within the surveyed area reveals significant differences in vegetation habitat characteristics. Specifically, the annual average precipitation in alpine meadow is markedly higher than that in alpine steppe (P< 0.05), and soil total organic carbon (TOC) levels are significantly greater in alpine meadow compared to alpine steppe (**Table 1**). These findings indicate that the climate in our study area trends towards aridification as we transition from alpine meadow to alpine steppe, accompanied by a loss of soil nutrients and a decline in land productivity. Such differences provide crucial environmental context for subsequent analyses regarding the impact of plateau pika interference on vegetation structure and function.

**Table 1:** Comparison of indexes of alpine steppe and alpine meadow.

Factors	AM	AS
Altitude	3515 ± 11.93^a^	3683 ± 60.44^b^
Annual mean temperature (°C)	1.05 ± 0.11^a^	-1.90 ± 0.27^a^
Average annual precipitation (mm)	660.71 ± 5.97^a^	154.92 ± 5.85^b^
pH	6.69 ± 0.08^a^	6.70 ± 0.16^a^
TOC	4.69 ± 1.09^a^	1.31 ± 0.08^b^
Bulk	1.38 ± 0.09^a^	1.44 ± 0.15^a^

This study examines the vegetation responses of alpine steppe and alpine meadow under varying plateau pika density disturbances and identifies significant differences in the sensitivity and recovery capabilities of the two ecosystems (**Table 2**). Although the alpine meadow shows clear negative changes in community height and the proportion of edible forage, its Shannon-Weiner diversity index, species richness, and aboveground biomass still exhibit a degree of recovery capacity. In contrast, the alpine steppe demonstrates negative changes across all indicators, particularly in Shannon-Weiner diversity index and species richness, suggesting that its ecosystem is more sensitive to plateau pika density disturbances, characterized by a simpler species composition and a more fragile ecological structure.

**Table 2:** Relative change rates of different indexes of two grassland types.

Grassland type	SW	SR	AB	CH	VC	HB
AM	54.14	25.68	101.53	-62.15	21.93	-36.98
AS	-36.20	-35.31	-29.89	-47.32	-29.05	-44.94

SW, Shannon-Weiner diversity index; SR, species richness; AB, aboveground biomass; CH, community height; VC, vegetation cover; HB, proportion of edible forage.

**Figure 2:**
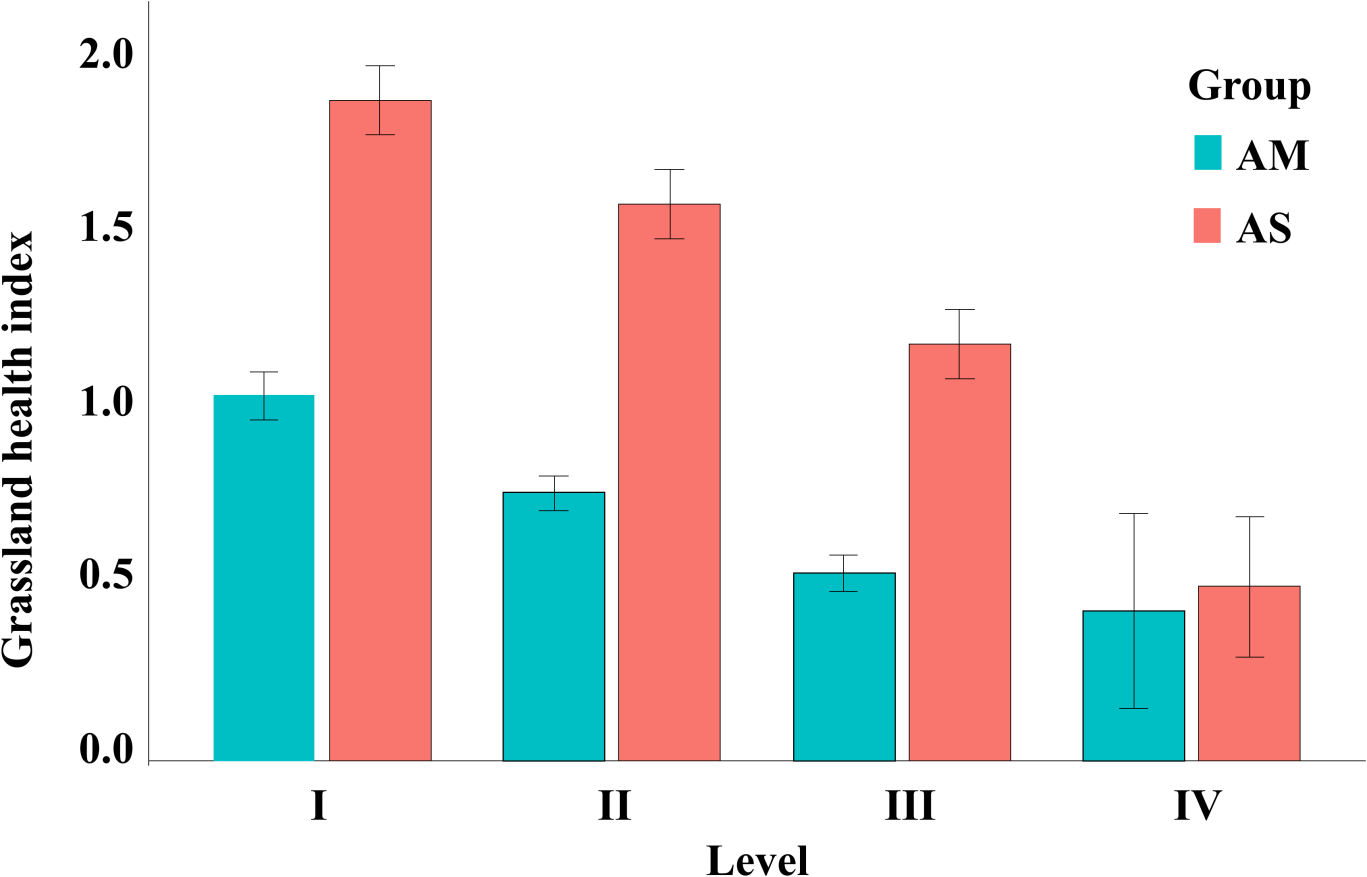
Disturbance degree classification of plateau pika.

The disturbance score GHI of plateau pika was calculated by using the principal component comprehensive evaluation method, which included vegetation cover, aboveground biomass, effective hole, community height, species richness, proportion of edible forage and Shannon-Wiener diversity index. The GHI is defined as the weighted sum of the above indicators. KMO (Kaiser-Meyer-Olkin) = 0.688 and Sig = 0.001 were obtained, indicating that the data met the analysis conditions. After calculating the comprehensive evaluation indicators of all sampling points, hierarchical clustering method was used to divide them into four levels (**Figure 2**): I (no significant disturbance), II (low disturbance), III (moderate disturbance) and IV (heavy disturbance).

### 3.2 Changes of vegetation communities under different disturbance levels of pika

**Figure 3:**
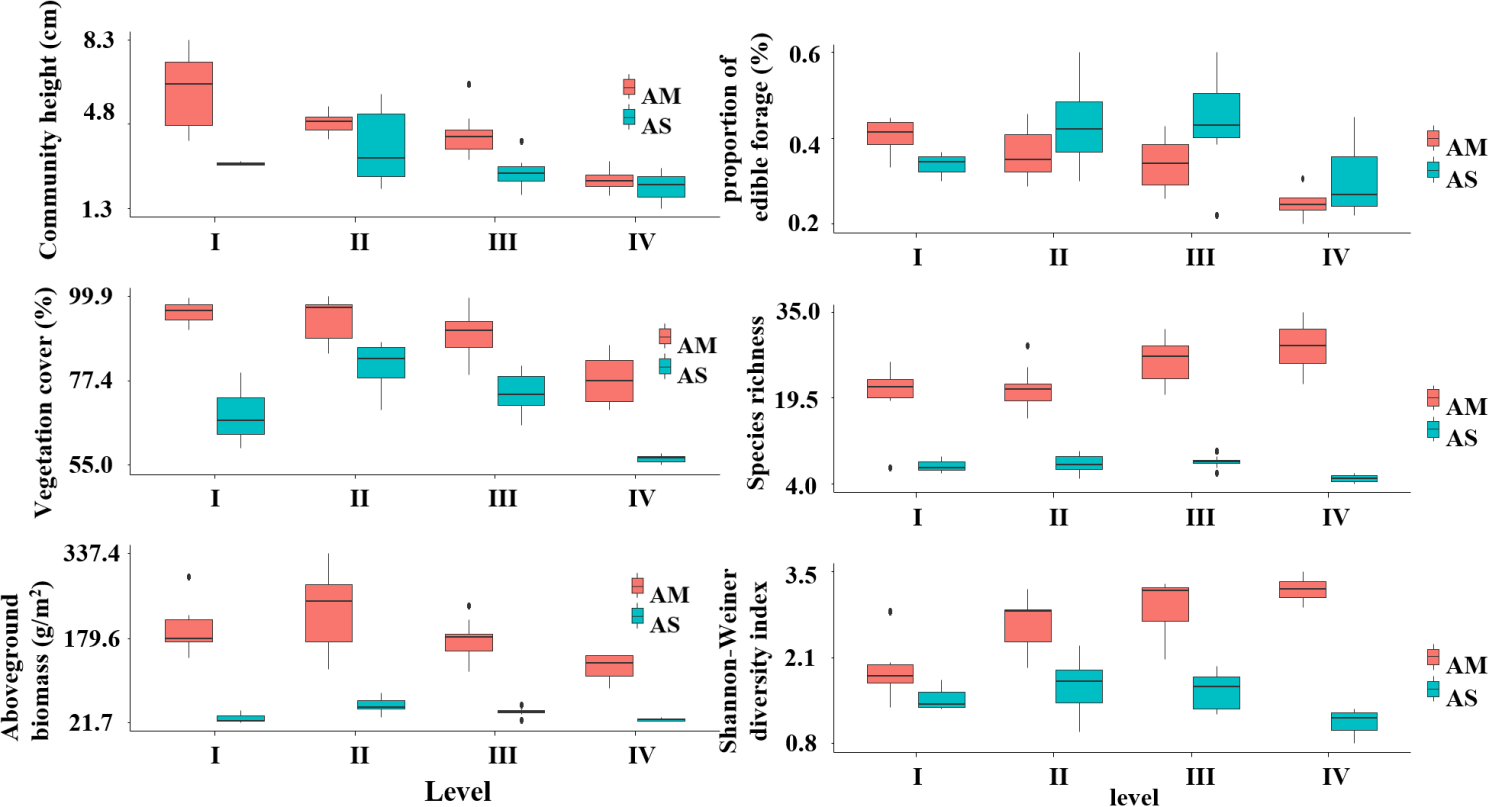
Vegetation community changes under different pika disturbance levels.

The results showed that in terms of community height, the vegetation height of both grassland types decreased with the increase of pika activity disturbance. The vegetation coverage of alpine meadow is the highest when there is no significant disturbance of pika, on the contrary, the vegetation coverage of alpine steep reaches its peak when the disturbance of pika is low disturbance. Aboveground biomass is highest for both alpine steppe and alpine meadow under moderate disturbance. The proportion of palatable forage is highest in the alpine meadow under low disturbance, while the alpine steppe shows peak values under moderate disturbance. For species richness and the Shannon-Wiener diversity index, the alpine steppe achieves its highest values under moderate disturbance, while the alpine meadow does so under low disturbance, displaying a trend of initially increasing and then decreasing with the gradient of plateau pika disturbance (**Figure 3**).

### 3.3 Changes of plant species richness under pika disturbance

**Figure 4:**
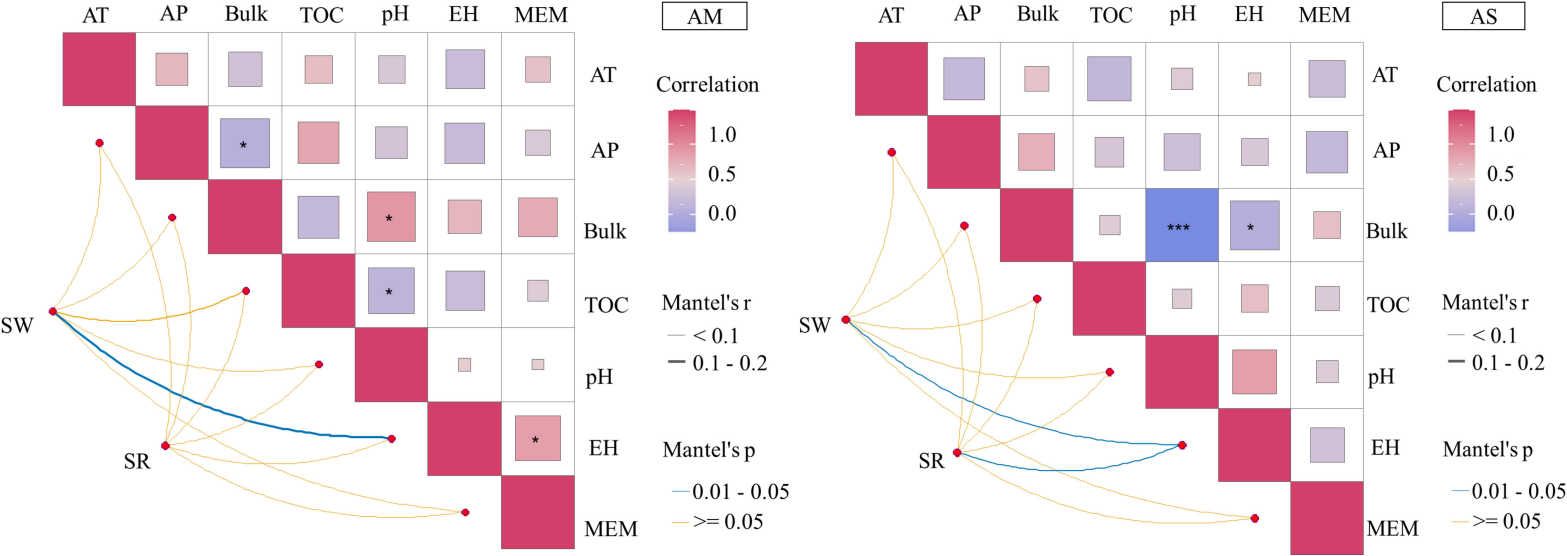
Shannon Wiener diversity and species richness in relation to other factors. AT, annual average temperature; AP, annual precipitation; TOC, total organic carbon; MEM, Moran’s eigenvector map; EH, effective hole; SW, Shannon-Weiner diversity index; SR, species richness.

Plant species richness and diversity index are important indicators to measure ecosystem health. Mantel test analysis showed that the Shannon-Wiener diversity of alpine meadow vegetation was significantly correlated with pika density (**Figure 4**; P< 0.05). The Shannon-wiener diversity index and species richness of in alpine steppe were significantly correlated with pika density (P< 0.05).

**Figure 5:**
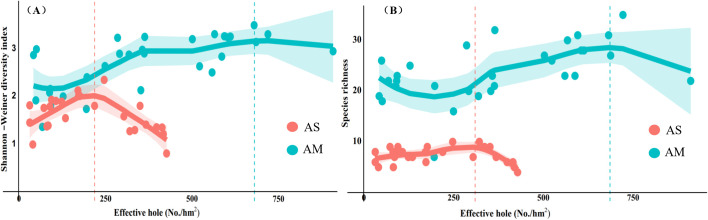
Response threshold of species diversity based on loess model. **(A)** shows the response thresholds of the Shannon-Wiener diversity index for two grassland types; **(B)** shows the response thresholds of species richness for two grassland types.

Loess regression analysis (**Figure 5**) revealed distinct response patterns between grassland types. In the alpine steppe, both species richness and the Shannon-Wiener diversity index exhibited early declines with increasing pika density, suggesting higher sensitivity to disturbance. In contrast, the alpine meadow maintained relatively stable biodiversity metrics until reaching significantly higher pika densities, demonstrating greater ecosystem resilience within a moderate disturbance range. Threshold analysis further quantified these differences. For the alpine steppe, peak biodiversity occurred at 217 holes/ha (Shannon index) and 307 holes/ha (species richness). To ensure comprehensive ecological protection, we adopted the more conservative threshold of 217 holes/ha, which safeguards both diversity dimensions before initial degradation. For the alpine meadow, the two metrics converged closely (680–683 holes/ha), supporting the selection of 680 holes/ha as a robust management threshold (**Table 3**).

**Table 3:** Threshold selection criteria.

Grassland type	Shannon-wiener threshold (effective holes/ha)	Species richness threshold (effective holes/ha)	Adopted threshold	Threshold selection principle
AS	217	307	217	Conservative value preserving both diversity dimensions
AM	680	683	680	Negligible difference (<0.5%) between metrics

**Figure 6:**
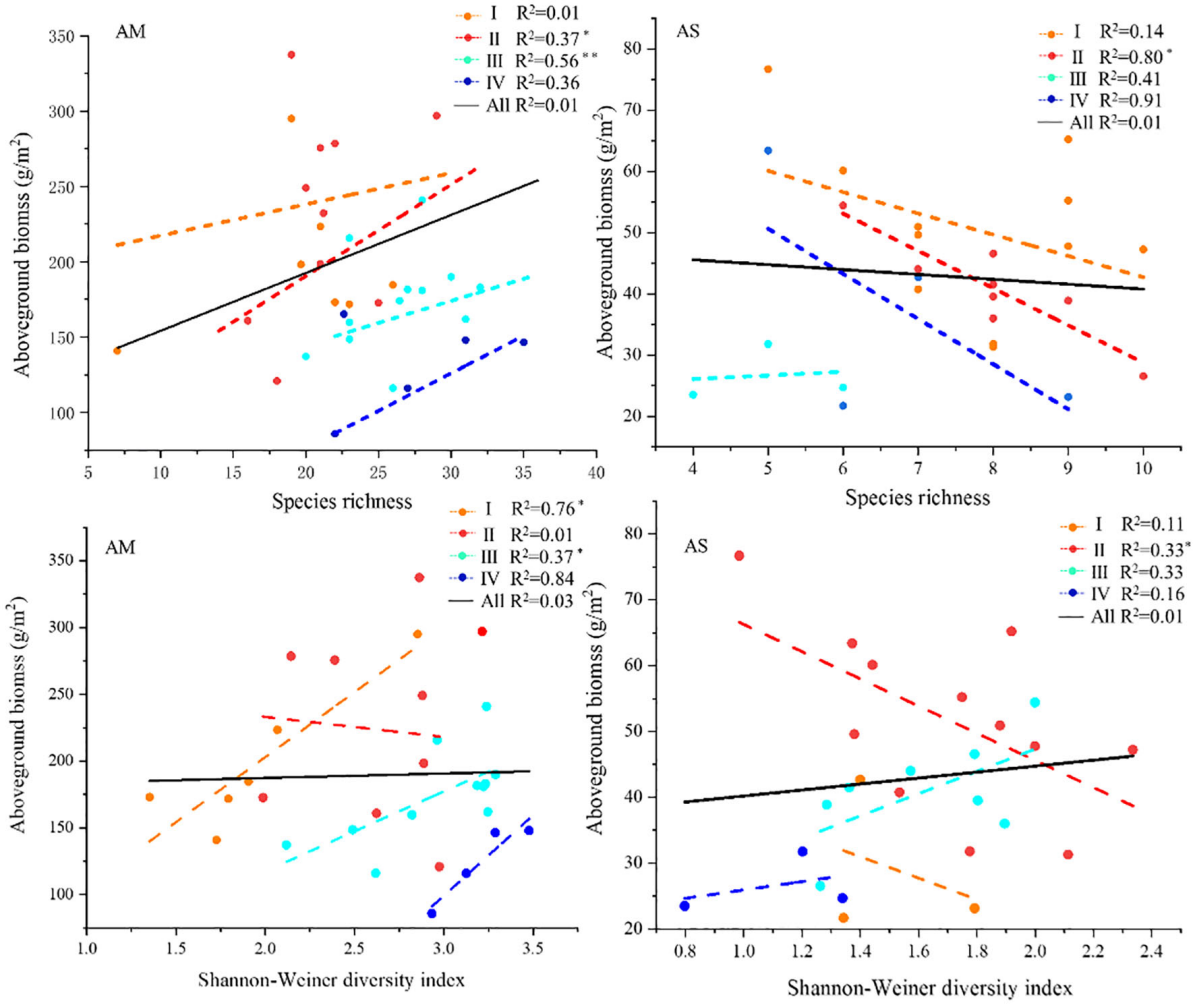
Relationship between diversity of different grassland types and aboveground biomass.

Through further analysis of the relationship between vegetation diversity and aboveground biomass, it was found that in alpine meadows, species richness and aboveground biomass were significantly positively correlated only under low and moderate disturbance, Shannon-Wiener diversity index was significantly positively correlated with aboveground biomass under no significant disturbance and negatively correlated with aboveground biomass under low disturbance. This indicates that vegetation communities with a certain degree of disturbance can maintain high biomass through rich species compensation mechanisms. For alpine steppe, species richness and Shannon-Wiener diversity index had significant negative effects on aboveground biomass only under low disturbance, which may be due to the stronger competition in alpine steppe, and the increase in species richness and diversity would lead to over-allocation of resources, thus inhibiting the accumulation of total biomass (**Figure 6**).

## 4 Discussion

Alpine steppe and alpine meadow are two typical grassland types on the Qinghai-Tibet Plateau, and they show significant differences in ecological functions such as productivity, diversity and ecological stability. These differences reflect the different response mechanisms of the two groups in the face of pika plateau interference, which leads to the possibility that the steppe community may be more sensitive to pika interference than the meadow.

### 4.1 Differences in sensitivity of different grassland types to pika disturbance

Alpine meadow shows higher ecological resilience, while alpine steppe shows degradation tendency under low disturbance. This difference may be due to the different ecological bases of the two grassland types in terms of water, nutrients and species diversity. The high precipitation and soil organic matter content of alpine meadows provide good recovery potential, enabling them to maintain high species diversity and functional stability under low and moderate disturbance (Grime, 2001). However, due to less precipitation and poorer soil conditions in the alpine steppe, plateau pika activity rapidly increase vegetation degradation, forming a “positive feedback” mechanism. Compared with alpine meadow, alpine steppe has relatively simple ecological structure and less functional groups and species composition in plant community (Tang et al., 2015). Lack of diversity may lead to functional redundancy (Delgado-Baquerizo et al., 2016). Therefore, plant populations in alpine steppes cannot effectively replace nibbled or destroyed species when disturbed by pika, resulting in significant declines in productivity, species diversity, and vegetation coverage (Hooftman, 1999). In addition, the soil and water conservation ability of alpine steppe is weak, and pika burrowing behavior further aggravates the damage of soil structure and limits the recovery of vegetation (Zhang et al., 2016). In general, although some ecological functions (such as vegetation height and edible forage ratio) of alpine meadow under pika interference showed certain degradation, its productivity and species diversity could recover well under moderate interference, showing strong ecological resilience. In contrast, alpine steppe, due to its low species diversity and weak ecological structure stability, showed a significant decline in productivity, poor resilience and higher sensitivity under pika interference. Therefore, in the future ecological protection and management, different measures should be taken for different grassland types, especially strengthening the monitoring and restoration management of alpine steppe, in order to deal with the potential risks brought by pika interference.

### 4.2 Effects of disturbance intensity on vegetation community and response threshold

It is found that above-ground biomass of the alpine meadow and the proportion of edible forage in the alpine steppe peak under moderate Disturbance, which is consistent with the intermediate disturbance hypothesis. Moderate disturbance of plateau pika may have reduced the competitive advantage of plant populations, allowing the high tolerance of edible forages and secondary functional groups to increase their relative abundance (Connell, 1978). For example, in areas moderately disturbed by plateau pika, above-ground biomass increased in alpine meadows, which may reflect that small-scale patches formed by pika activity promoted heterogeneity in photosynthesis and nutrient access, while this promotion was quickly masked by community degradation under high intensity disturbance.

Under plateau pika disturbance, significant differences in ecological responses were observed between grassland types, with the alpine steppe exhibiting markedly lower Shannon-Wiener diversity index and species richness compared to the alpine meadow. The meadow system maintained relatively high species diversity and aboveground biomass until reaching higher disturbance intensities, indicating an elevated disturbance threshold prior to ecological function loss. In contrast, the steppe ecosystem demonstrated greater vulnerability due to its simpler vegetation structure and limited productivity. These findings align with the stability-diversity hypothesis (Bennett et al., 2020), suggesting that the meadow’s richer plant species composition and more complex ecological structure confer greater resistance to pika disturbance compared to the more fragile steppe ecosystem (Tilman et al., 1997). The identified management thresholds (217 burrows/ha for steppe versus 680 burrows/ha for meadow) reflect these fundamental ecological differences, necessitating distinct conservation approaches: stringent pika control in the sensitive alpine steppe to prevent biodiversity loss, versus more tolerant management in meadows that accommodates the ecological benefits of moderate pika activity. These results underscore the importance of ecosystem-specific management strategies that balance biodiversity conservation with recognition of plateau pikas’ ecological role. Future research should focus on elucidating the underlying mechanisms of these divergent resilience patterns, particularly plant-soil feedback systems, to refine predictive management models and establish more precise management thresholds.

In addition, we also analyzed the relationship between species richness and biomass. In alpine meadow, species richness was positively correlated with aboveground biomass only under mild and moderate disturbance. In alpine steppe, above-ground biomass decreased with the increase of plant species richness, and no significant relationship was observed at moderate and above disturbance levels. This may be because plant communities in alpine meadows are usually more complex in structure, and there may be strong functional compensation between species (de Silva et al., 2021). For example, under mild and moderate disturbance, some species with high tolerance may fill the ecological niche of the damaged species, thereby maintaining the overall biomass. That is, moderate disturbance may enhance the association between species diversity and functional performance by increasing habitat heterogeneity and promoting resource allocation optimization (Connell, 1978). The plant community of alpine steppe is relatively homogeneous, and the plants may face strong competitive pressure. When species richness increases, competition between plants may result in inhibited growth of some species, thereby reducing overall above-ground biomass.

### 4.3 Implications of different interference levels for management

This study further verified the hypothesis that the response threshold of pika interference in alpine steppe is earlier than that in alpine meadow. This early response suggests that alpine steppe ecosystems might be more sensitive to pika disturbance under current conditions. Future studies should explore how climate change interacts with pika populations to further assess ecosystem vulnerability. In practice, the time window of grassland restoration work needs to be precisely planned according to the response threshold of grassland type to avoid irreversible ecological degradation. From the perspective of grassland management, differentiated measures should be taken for different grassland types. For alpine meadows, management should focus on maintaining moderate disturbance levels, such as maintaining community diversity and productivity through a combination of green control by introducing natural enemies and artificial vegetation restoration. For the alpine steppe, management should focus on maintaining low disturbance levels, pika interference intensity should be reduced as much as possible to avoid its rapid triggering of degradation threshold.

The results demonstrate significant differences in disturbance thresholds of plateau pika activities between alpine meadows and alpine steppe. These findings suggest that current unified management standards for pika population density may need reconsideration to account for the differing ecosystem sensitivities across Qinghai-Tibet Plateau grasslands. The study particularly highlights alpine steppe’s greater vulnerability to low-intensity disturbances, indicating that more targeted protection strategies could improve high-altitude grassland management and restoration efforts.

## Data Availability

The original contributions presented in the study are included in the article/**Supplementary Material**. Further inquiries can be directed to the corresponding authors.
