# Supplementary material for: Alpine steppe vegetation communities are more sensitive to plateau pika disturbance than alpine meadows
Source: Front Plant Sci. 2025 May 29;16:1546828. doi: 10.3389/fpls.2025.1546828 (PMC12159023; doi:10.3389/fpls.2025.1546828)
Supplement: Supplementary file 1 [file DataSheet1.docx]

Supplementary Material

**Alpine Steppe Vegetation Communities Are More Sensitive to Plateau Pika Disturbance Than Alpine Meadows**

# Rui Hua1, Peng Zhang2, Liqing Wang1, Miaomiao Huang3, Limin Hua1*, JianWei Zhou1*

# Supplementary Figures

**
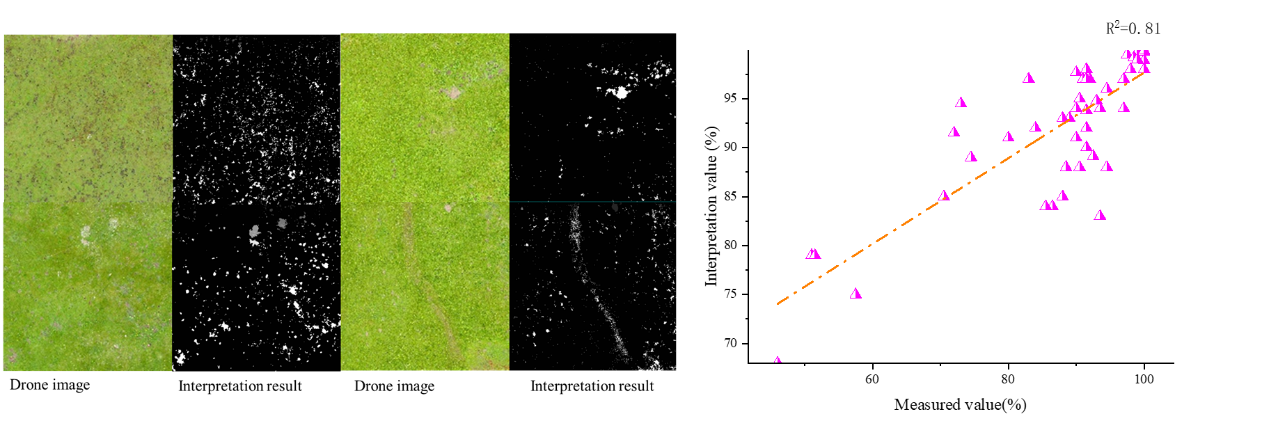
**

**Supplementary Figure S1.** Analysis of measured values and interpretation values of unmanned aerial vehicle images
